# New Insights into the Formation of Viable but Nonculturable *Escherichia coli* O157:H7 Induced by High-Pressure CO_2_

**DOI:** 10.1128/mBio.00961-16

**Published:** 2016-08-30

**Authors:** Feng Zhao, Yongtao Wang, Haoran An, Yanling Hao, Xiaosong Hu, Xiaojun Liao

**Affiliations:** a Beijing Advanced Innovation Center for Food Nutrition and Human Health, Beijing, China; b College of Food Science and Nutritional Engineering, China Agricultural University, Beijing, China; c Key Lab of Fruit and Vegetable Processing, Ministry of Agriculture, Beijing, China

## Abstract

The formation of viable but nonculturable (VBNC) *Escherichia coli* O157:H7 induced by high-pressure CO_2_ (HPCD) was investigated using RNA sequencing (RNA-Seq) transcriptomics and isobaric tag for relative and absolute quantitation (iTRAQ) proteomic methods. The analyses revealed that 97 genes and 56 proteins were significantly changed upon VBNC state entry. Genes and proteins related to membrane transport, central metabolisms, DNA replication, and cell division were mainly downregulated in the VBNC cells. This caused low metabolic activity concurrently with a division arrest in cells, which may be related to VBNC state formation. Cell division repression and outer membrane overexpression were confirmed to be involved in VBNC state formation by homologous expression of *z2046* coding for transcriptional repressor and *ompF* encoding outer membrane protein F. Upon VBNC state entry, pyruvate catabolism in the cells shifted from the tricarboxylic acid (TCA) cycle toward the fermentative route; this led to a low level of ATP. Combating the low energy supply, ATP production in the VBNC cells was compensated by the degradation of l-serine and l-threonine, the increased AMP generation, and the enhanced electron transfer. Furthermore, tolerance of the cells with respect to HPCD-induced acid, oxidation, and high CO_2_ stresses was enhanced by promoting the production of ammonia and NADPH and by reducing CO_2_ production during VBNC state formation. Most genes and proteins related to pathogenicity were downregulated in the VBNC cells. This would decrease the cell pathogenicity, which was confirmed by adhesion assays. In conclusion, the decreased metabolic activity, repressed cell division, and enhanced survival ability in *E. coli* O157:H7 might cause HPCD-induced VBNC state formation.

## IMPORTANCE

*Escherichia coli* O157:H7 has been implicated in large foodborne outbreaks worldwide. It has been reported that the presence of as few as 10 cells in food could cause illness. However, the presence of only 0.73 to 1.5 culturable *E. coli* O157:H7 cells in salted salmon roe caused infection in Japan. Investigators found that *E. coli* O157:H7 in the viable but nonculturable (VBNC) state was the source of the outbreak. So far, formation mechanisms of VBNC state are not well known. In a previous study, we demonstrated that high-pressure CO_2_ (HPCD) could induce the transition of *E. coli* O157:H7 into the VBNC state. In this study, we used RNA-Seq transcriptomic analysis combined with the iTRAQ proteomic method to investigate the formation of VBNC *E. coli* O157:H7 induced by HPCD treatment. Finally, we proposed a putative formation mechanism of the VBNC cells induced by HPCD, which may provide a theoretical foundation for controlling the VBNC state entry induced by HPCD treatment.

## INTRODUCTION

A viable but nonculturable (VBNC) state has been widely observed in many bacteria (1), and many kinds of stresses can induce its formation (2). Colwell noted that the VBNC state may represent a dormant state that improved the survival of nonsporulating bacteria under adverse environmental conditions (3). Under appropriate conditions, bacteria in the VBNC state can be restored to the culturable state (2), which would cause economic loss and pose a health risk. High-pressure CO_2_ (HPCD), one of nonthermal pasteurization techniques, is an effective means to inactivate microorganisms in foodstuffs and medicines. The effects of HPCD pasteurization on microorganisms have been ascribed to the interaction of anaerobic conditions, acidification, pressure, and high CO_2_ concentrations (4). In a previous study, we demonstrated for the first time that HPCD could induce the transition of *Escherichia coli* O157:H7, a pathogenic bacterium, into the VBNC state (5), which poses a potential health risk for HPCD-treated products. In order to control the VBNC state entry induced by HPCD, it is very important to investigate the formation mechanisms of VBNC bacteria.

Until now, studies on the VBNC state have mainly focused on its induction, resuscitation, and physiological properties. In recent years, molecular characteristics of VBNC bacteria which could help reveal formation mechanisms of the VBNC state have been investigated. Kong et al. (6) found that cold-induced loss of culturability in *Vibrio vulnificus* occurred concomitantly with the loss of catalase activity. This phenomenon was also observed in VBNC *Staphylococcus aureus* (7). Asakura et al. (8) reported that negative modulation of RNA polymerase sigma S (RpoS) expression could promote the VBNC state formation in *E. coli* O157:H7 under conditions of osmotic and oxidative stresses. However, those studies focused on a few specific genes and proteins, which can reflect formation mechanisms of the VBNC state only partly. Using two-dimensional electrophoresis (2-DE) proteomic analysis, Heim et al. (9) found that the protein profile of VBNC *Enterococcus faecalis* was different from that of starved or exponential-phase cells. Meanwhile, differentially expressed proteins involved in many metabolic pathways were identified in the VBNC state of *Brettanomyces* (10) and *Vibrio parahaemolyticus* (11) by 2-DE analysis, and Serpaggi et al. (10) proposed that oxidative stress would be a driving factor for VBNC state entry. Furthermore, a comparative microarray study has revealed genes differentially expressed between VBNC and unstressed *V. cholerae* cells (12). Using RNA sequencing (RNA-Seq), Su et al. (13) pointed out that the presence of the up- and downregulated genes involved in ATP accumulation, protein modification, and membrane proteins would benefit the survival of VBNC *Rhodococcus biphenylivorans* under oligotrophic and low-temperature conditions. However, those studies mainly focused on the metabolic features of the VBNC state rather than on VBNC state formation; meanwhile, they lacked functional verification for transcriptomic or proteomic analyses.

Next-generation sequencing (NGS) technology, i.e., RNA-Seq, can produce more in-depth information, such as information on low-abundance transcripts, as it provides higher resolution and higher sensitivity than microarray methods (14). Additionally, the isobaric tag for relative and absolute quantitation (iTRAQ) proteomic technique enables global protein identification and quantification with no molecular weight and solubility limitations (15). In this study, RNA-Seq transcriptomics combined with the use of the iTRAQ proteomic method was performed to analyze differentially expressed genes and proteins in VBNC *E. coli* O157:H7 induced by HPCD treatment and to provide new insights into VBNC state formation. Finally, a putative mechanism of VBNC state formation induced by HPCD is proposed in this report.

## RESULTS AND DISCUSSION

**FIG 1:**
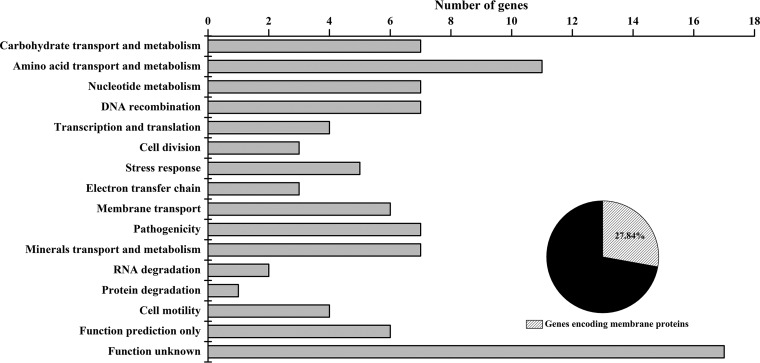
The number of differentially expressed genes in VBNC cells assigned to the 14 biological processes. Genes were categorized according to the function of their encoded proteins from the KEGG pathways for *Escherichia coli* O157:H7 EDL933. The pie chart indicates the percentage of genes coding for membrane proteins compared to the total differentially expressed genes.

High-throughput Illumina RNA-seq was used to investigate the transcriptome level changes in the VBNC cells and the control. After filtering dirty raw reads, a total of 25,674,506 and 25,707,052 reads were obtained for the VBNC cells and the control, respectively. Approximately 95% and 92% of total reads for the VBNC cells and the control were uniquely mapped to the reference genome, respectively (see Table S1 in the supplemental material). Additionally, the total number of mapped reads for the VBNC cells and the total number of mapped reads for the control were distributed homogeneously in the reference genes (see Fig. S1 in the supplemental material), suggesting that the reads obtained by RNA-seq were generated from the whole genes rather than from specific regions in the genes. Through mapping to the reference genome, 4,651 and 4,653 transcripts were identified for the VBNC cells and the control, respectively, and gene coverage of these transcripts is shown in Fig. S2. According to the screening criteria for differentially expressed genes, the transcription of 97 genes, including 22 upregulated genes and 75 downregulated genes, was found to be associated with the VBNC state. These differentially expressed genes were classified in a variety of functional categories (Fig. 1), as detailed in Table S2.

**FIG 2:**
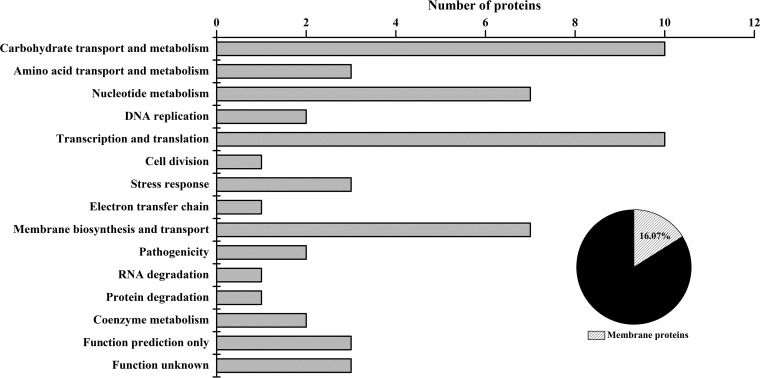
The number of differentially expressed proteins in VBNC cells assigned to the 13 biological processes. The pie chart indicates the percentage of membrane proteins compared to the total differentially expressed proteins.

In addition, the iTRAQ method was performed to identify the proteins that were differentially expressed between the VBNC cells and the control. A total of 7,194 unique peptides associated with 1,573 proteins were identified (see Table S3 in the supplemental material). The molecular masses of these proteins were mainly in the range of 10 to 60 kDa (see Fig. S3), and functional classification of the total identified proteins was performed by COG analysis (see Fig. S4). Among the identified proteins, the abundance of 56 was significantly changed by a factor of >1.2-fold (*P* < 0.05), the protein functions were classified as shown in Fig. 2, and detailed information about every protein is listed in Table S4. Among these proteins, 28 proteins were upregulated and 28 proteins were downregulated. As these differentially expressed genes and proteins may be related to VBNC state formation under HPCD treatment conditions, we discuss them in detail as follows.

### Central metabolic processes. (i) Carbohydrate transport and metabolism.

In VBNC cells, some genes and proteins related to the phosphoenolpyruvate:carbohydrate phosphotransferase system (PTS) were downregulated, including enzyme I of PTS encoded by *ptsI* and three genes (*agaD*, *srlA_1*, and *ulaA*) encoding enzyme II CD components for different PTSs. In addition, transcription levels of *lamB* and *malM*, involved in maltose assimilation, were also downregulated in the VBNC cells. These results indicate that the carbohydrate transport activity was reduced upon VBNC state entry.

Glucose-6-phosphate dehydrogenase (Zwf) and 6-phosphogluconate dehydrogenase (Gnd), which are involved in converting glucose-6-phosphate into ribulose-5-phosphate concurrently with NADPH generation, were 1.62- and 1.33-fold more abundant in the VBNC cells. Regeneration of NADPH is a cellular response of *E. coli* to oxidative stress (16); thus, overproduction of Zwf and Gnd was probably an *E. coli* O157:H7 tolerance response to oxidative stress arising from acid stress (17, 18) and high pressure (19) caused by HPCD treatment.

In the VBNC cells, d-lactate dehydrogenase encoded by *ldhA*, which is responsible for fermentative conversion of pyruvate to d-lactate, was 1.53-fold more abundant, but the amount of another d-lactate dehydrogenase encoded by *dld*, which catalyzes the conversion of d-lactate to pyruvate, was decreased by 4.76-fold. The differential expression levels of LdhA and Dld indicate that overproduction of d-lactate occurred upon VBNC state formation. In addition, the amounts of the E1 subunit of the pyruvate dehydrogenase complex (AceE) and citrate synthase (GltA), which catalyze pyruvate to form citrate, were increased by 1.51- and 1.85-fold, respectively. However, the abundance of LipA, which catalyzes the lipoyl cofactor formation, was decreased in the VBNC cells. As the lipoyl cofactor is required for the activity of the pyruvate dehydrogenase complex (20), a decreased amount of LipA would repress the activity of pyruvate dehydrogenase complex in the VBNC cells. Meanwhile, the activity of GltA can be inhibited by an excess of NADPH produced in the VBNC cells (21). Furthermore, the decreased abundance of AcnB, which catalyzes isomerization of citrate into isocitrate, was detected after HPCD tretatment. These results suggest that the carbon flux occurring through the tricarboxylic acid (TCA) cycle was decreased upon VBNC state entry, which would reduce ATP and CO_2_ production. Using 2-DE analysis, Serpaggi et al. (10) also found that the level of glycolytic flux was reduced in SO_2_-induced VBNC *Brettanomyces*. During formation of the VBNC state, pyruvate catabolism shifted from the TCA cycle to the fermentative route would depress the cell growth rate.

### (ii) Amino acid transport and metabolism.

In VBNC cells, transcript levels of genes that participated in arginine transport and biosynthesis (*artJ*, *argC*, and *argI*) and methionine transport and biosynthesis (*metN*, *metA*, and *metF*) were downregulated. Meanwhile, transcription of *metR*, which encodes a transactivator of transcription of *metE* and *metH*, which catalyze the final step in methionine biosynthesis, was also downregulated. These results suggest that the ability of arginine and methionine to perform transport and biosynthesis was reduced upon VBNC state entry, leading to the decreased protein synthesis rate.

It is worth noting that proteins and genes related to biosynthesis and degradation of l-serine (SerA, 1.49-fold; *z4464*, 2.30-fold) and l-threonine (ThrC, 2.23-fold; *tdcB*, 2.00-fold; *yhaR*, 2.16-fold) were upregulated in the VBNC cells, although the upregulation of *tdcB* was statistically insignificant. Hesslinger et al. (22) reported that catabolism of l-serine and l-threonine provided *E. coli* cells with a source of energy under anaerobic conditions; thus, enhancement of production and degradation of l-serine and l-threonine is probably an energy generation mode upon VBNC state entry under the anaerobic conditions caused by HPCD treatment. In addition, ammonia produced during the degradation of l-serine and l-threonine can help *E. coli* O157:H7 cells resist acid stress caused by HPCD treatment.

### (iii) Nucleotide metabolism.

PurF, PurD, and PurL, which are involved in the *de novo* purine biosynthesis pathway leading from phosphoribosylpyrophosphate (PRPP) to IMP, were 4.33-, 1.49-, and 1.67-fold less abundant under HPCD treatment conditions, respectively, although the reductions in the amounts of PurF and PurD were statistically insignificant. These results suggest that the amount of IMP is decreased in the VBNC cells. IMP is a branch point metabolite which can be converted to adenine and guanine nucleotides (23). In the VBNC cells, the amount of GuaB catalyzing the first reaction in the *de novo* pathway of synthesis of GMP from IMP was decreased. However, the abundance of PurA, which mediates conversion of IMP to AMP, was increased. These results indicate that IMP was mainly converted to AMP upon VBNC state entry and might be further converted to ATP. Parry and Shain (24) proposed that elevated ATP levels in *E. coli* appeared to represent a global response to decreased reaction rates; thus, the trend toward increasing ATP levels was probably a response to the low metabolic activity of the VBNC state. Su et al. (13) also found that genes related to ATP biosynthesis were upregulated in VBNC *R. biphenylivorans*. Decreased abundance of the large subunit of carbamoyl phosphate synthase (encoded by *carB*), which initiates *de novo* pyrimidine biosynthesis, was found in the VBNC cells, which would decrease the level of pyrimidine nucleotides. In conclusion, the decreased amounts of purine and pyrimidine nucleotides would inhibit the synthesis of DNA and RNA, which may be a reason for the loss of culturability upon VBNC state entry.

Based on the results described above, it was found that the central metabolic activity in *E. coli* O157:H7 was decreased upon VBNC state formation. Through enzymatic activity analysis by the use of an API ZYM kit, the activities of all enzymes in HPCD-induced VBNC cells were shown to be lower than those in the exponential-phase cells (5). As enzymes are essential to metabolic processes, the result of enzymatic activity in the VBNC cells confirmed the results of the transcriptomic and proteomic analyses described above.

### Electron transfer chain.

HemL, which catalyzes the transamination of glutamate-1-semialdehyde to 5-aminolevulinic acid (ALA), the common precursor of all tetrapyrroles, including heme, was 1.99-fold more abundant in the VBNC cells. Thus, overproduction of HemL would enhance the content of heme in the VBNC cells. In addition, the transcription level of *ccmD*, encoding heme exporter protein D, which is involved in heme delivery to the heme chaperone during cytochrome *c* maturation, was upregulated 2.21-fold in the VBNC cells. Therefore, overexpression of HemL and *ccmD* would increase the amount of cytochrome *c* and thus might enhance electron transfer in VBNC cells. Meanwhile, transcript levels of *yodB* (coding for cytochrome *b561*) and *z2702* (encoding YdhY) were upregulated 2.41-fold and 2.10-fold, respectively. Cytochrome *b561* is an electron-transferring component of the respiratory chain (25). YdhY is predicted to be a ferredoxin-like protein and is possibly involved in electron transfer reactions (26). These results suggest that the ability to transfer electrons was enhanced upon VBNC state formation, which might increase the ATP level and thus provide energy for the survival of the cell.

### Gene replication and expression. (i) DNA replication and recombination.

In the VBNC cells, a 2.78-fold decrease in the amount of DnaE, which contains the 5′–3′ DNA polymerase activity, was detected which would suppress elongation of DNA replication. Moreover, SeqA, a major negative regulator of the onset of DNA replication, was 1.76-fold more abundant in the VBNC cells, which would restrain replication initiation. Therefore, DNA replication was inhibited in *E. coli* O157:H7 cells by suppressing its initiation and elongation upon VBNC state formation. Furthermore, absence of SeqA in *E. coli* leads to formation of overly condensed and supercoiled nucleoids and purified SeqA generates positive DNA supercoils *in vitro* (27, 28). Using transmission electron microscopy (TEM), we found that the nucleoid structure was loosened in the VBNC cells (5), which corresponds to the fact of SeqA overproduction upon VBNC state entry.

At the transcriptome level, two genes, *z2981* and *z5490*, involved in transposition recombination, were downregulated 2.56- and 2.22-fold in the VBNC cells, respectively. This result suggests that the activity of transposition recombination in *E. coli* O157:H7 cells was decreased upon VBNC state entry.

### (ii) Transcription and translation.

The amount of DNA topoisomerase I (TopA) was increased by 1.88-fold in the VBNC cells. TopA can remove hypernegative supercoils generated on the DNA during transcription elongation (29). DNA supercoiling is considered to be one link between environmental changes and gene expression (30). Weinstein-Fischer et al. (30) proposed that *topA* activation in *E. coli* leads to DNA relaxation and possibly to selective gene expression, which was important for the response to oxidative stress. Thus, the cellular level of TopA may be involved in stress response by affecting gene expression. In addition, the abundance of transcription elongation factor GreA was increased by 1.64-fold in the VBNC cells. GreA is important for transcriptional regulation under conditions of stresses (31). Therefore, overproduction of TopA and GreA could ensure that transcription elongation proceeds smoothly, which would enhance the survival of *E. coli* O157:H7 cells in response to HPCD during VBNC state entry.

Amounts of 30S ribosomal proteins S1 (RpsA) and S5 (RpsE) involved in translation initiation and fidelity were decreased in the VBNC cells, suggesting that the translational ability of *E. coli* O157:H7 cells was reduced upon VBNC state formation. Additionally, the amount of the α subunit of glycyl-tRNA synthetase (GlyQ) was decreased in the VBNC cells, but isoleucyl-tRNA synthetase (IleS) and glutaminyl-tRNA synthetase (GlnS) were overproduced. This result suggests that selective protein translation proceeds upon VBNC state formation, which would enhance the survival of *E. coli* O157:H7 cells in response to HPCD treatment. However, 30S ribosomal protein S4 (RpsD) and 50S ribosomal protein L24 (RplX), responsible for the assembly of 30S and 50S ribosomal subunits, were 1.39- and 1.55-fold more abundant in the VBNC cells, respectively. This would promote assembly of ribosomes upon VBNC state entry. By TEM analysis, we found that ribosomes in the VBNC cells were dissociated (5), which accorded with the decreased translational ability shown in the proteomic analysis. Meanwhile, upregulation of ribosomal assembly proteins would stimulate reassembly of these dissociated ribosomes, which might benefit the survival of *E. coli* O157:H7 cells during VBNC state formation.

Taking the data together, the overall activity of gene replication and expression was decreased upon VBNC state entry. But for survival, selective transcription and translation were retained in the cells.

### Cell division.

**FIG 3:**
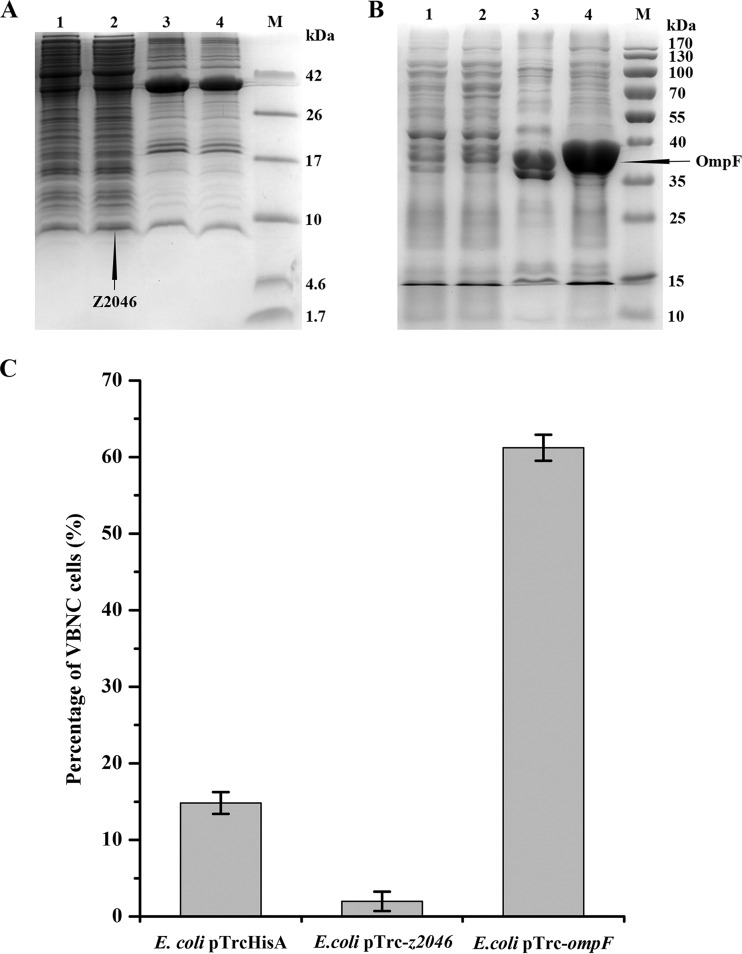
VBNC state formation for recombinant *Escherichia coli* O157:H7 strains upon HPCD treatment. (A and B) Analysis of homologous expression of *z2046* and *ompF* upon 1 mM IPTG induction using Tricine–SDS-PAGE and SDS-PAGE, respectively. Lanes 1, soluble fraction of *E. coli* pTrcHisA; lanes 2, soluble fraction of *E. coli* pTrc-*z2046* (A) and *E. coli* pTrc-*ompF* (B); lanes 3, insoluble fraction of *E. coli* pTrcHisA; lanes 4, insoluble fraction of *E. coli* pTrc-*z2046* (A) and *E. coli* pTrc-*ompF* (B); lanes M, marker. (C) The percentage of VBNC cells for the control, *E. coli* pTrc-*z2046* and *E. coli* pTrc-*ompF* treated by HPCD at 5 MPa and 25°C for 15 min. Error bars represent standard deviation (SD).

Transcript levels of *z1876* and *z2371*, both encoding lysozyme, were downregulated 2.70- and 6.67-fold in the VBNC cells, respectively. This would reduce the rate of cell autolysis and thus maximize the proportion of viable cells upon VBNC state entry. DamX, one of division proteins, was 1.85-fold more abundant in the VBNC cells. When overproduced, DamX inhibits cell division (32); thus, increased amounts of DamX in *E. coli* O157:H7 cells would repress cell division upon VBNC state formation. Furthermore, 20.00-fold downregulation of the *z2046* gene coding for DicC was observed in the VBNC cells. DicC is a transcriptional repressor of the division inhibition gene *dicB* (33), so downregulation of *z2046* would derepress expression of *dicB*, leading to the cell division inhibition. In this study, a recombinant *E. coli* O157:H7 strain harboring *z2046* was constructed. Upon IPTG (isopropyl-β-d-thiogalactopyranoside) induction, an expected 8.3-kDa protein was overproduced in the soluble fraction of *E. coli* pTrc-*z2046* (Fig. 3A). Combined with liquid chromatography-tandem mass spectrometry (LC-MS/MS) analysis, it was demonstrated that *z2046* was successfully expressed in the recombinant strain. After HPCD treatment, the percentage of VBNC cells for the IPTG-induced *E. coli* pTrc-*z2046* strain was about 7.46-fold lower than that for the control strain (Fig. 3C), indicating that overexpression of *z2046* suppressed VBNC state formation. This result confirmed the result of the transcriptomic analysis, i.e., that downregulation of *z2046* in *E. coli* O157:H7 would be conducive to VBNC state entry induced by HPCD treatment. These results suggest that differentially expressed DamX and *z2046* can cause a loss of culturability in *E. coli* O157:H7 cells during VBNC state formation.

### Membrane biosynthesis.

In the VBNC cells, the abundance of GalU was increased by 2.10-fold. GalU catalyzes the production of UDP-glucose, an important glucosyl donor in lipopolysaccharide (LPS) biosynthesis (34). Thus, overexpression of GalU upon VBNC state entry would contribute to maintain the outer membrane integrity in *E. coli* O157:H7 cells. Additionally, sn-glycerol-3-phosphate dehydrogenase (GpsA) was 1.59-fold more abundant in the VBNC cells, but a 2.33-fold decrease in the amount of sn-glycerol-3-phosphate dehydrogenase subunit A (GlpA) was detected. GpsA is involved in sn-glycerol-3-phosphate synthesis (35), while GlpA catalyzes the oxidization of sn-glycerol-3-phosphate to dihydroxyacetone-phosphate (DHAP) (36). In *E. coli*, sn-glycerol-3-phosphate is a direct precursor for phospholipid biosynthesis (37). Therefore, the differential expression of GpsA and GlpA would increase the amount of phospholipid, which can participate in maintaining cell membrane integrity during VBNC state formation. Moreover, HPCD treatment can cause a loss of phospholipid in *E. coli* membrane (38), so the increase in the content of phospholipid may increase the survival of *E. coli* O157:H7 cells under conditions of HPCD treatment.

The expression of most membrane proteins was repressed in the VBNC cells, but that of several membrane proteins was upregulated, including outer membrane protein F (OmpF) (1.71-fold upregulation), one of the most abundant outer membrane proteins in *E. coli* (39). Asakura et al. (40) also found that OmpW was overproduced in VBNC *E. coli* O157:H7 cells. In *E. coli*, OmpF expression is regulated by the EnvZ-OmpR two-component system. Darcan et al. (41) pointed out that EnvZ played an important role in triggering VBNC state entry in *E. coli*. This indicates that OmpF might participate in VBNC state formation. A recombinant *E. coli* O157:H7 strain harboring *ompF* was constructed in this study, and an expected 39.4-kDa protein was obtained in the insoluble fraction of the recombinant strain upon IPTG induction (Fig. 3B). LC-MS/MS analysis showed that *ompF* was successfully expressed in the recombinant strain. After HPCD treatment, the proportion of VBNC cells seen with the IPTG-induced *E. coli* pTrc-*ompF* strain was 61.22%, which was about 4.13-fold higher than the proportion of VBNC cells seen with the control strain (Fig. 3C), suggesting that overproduction of OmpF promoted VBNC state entry. But the exact molecular mechanism of VBNC state entry caused by OmpF is still unknown. In *E. coli*, overexpression of OmpF activates the alternative sigma factor E (RpoE), which controls an envelope stress-responsive system (42, 43). Perhaps envelope stress response caused by OmpF leads to VBNC state formation.

Although the content of phospholipid and membrane proteins changed in the VBNC cells, fluorescence anisotropy measurement showed that there was no significant difference (*P* > 0.05) between the fluorescence polarization of the VBNC cells and that of the control (see Fig. S5 in the supplemental material). This result suggests that the membrane fluidity did not change when the cells entered the VBNC state. Membrane fluidity maintenance in the VBNC cells could partially demonstrate the membrane integrity, which fits the membrane characteristics of VBNC bacteria (40). Meanwhile, membrane fluidity retention would help retain membrane functions, which may favor VBNC cell survival.

### General stress response.

In the VBNC cells, some genes and proteins that participate in general stress responses were upregulated, including molecular chaperone GroEL, heat shock protein 90 (Hsp90), *hdeA*, and *hdeB*. In *E. coli*, GroEL assists the folding of nascent or stress-denatured proteins (44), and Hsp90 promotes client-protein folding and stabilization (45). Moreover, HdeA and HdeB in *E. coli* can prevent the aggregation of periplasmic proteins at acidic pH (pH <3) (46, 47). These upregulated molecular chaperons would facilitate cell survival during VBNC state entry.

**FIG 4:**
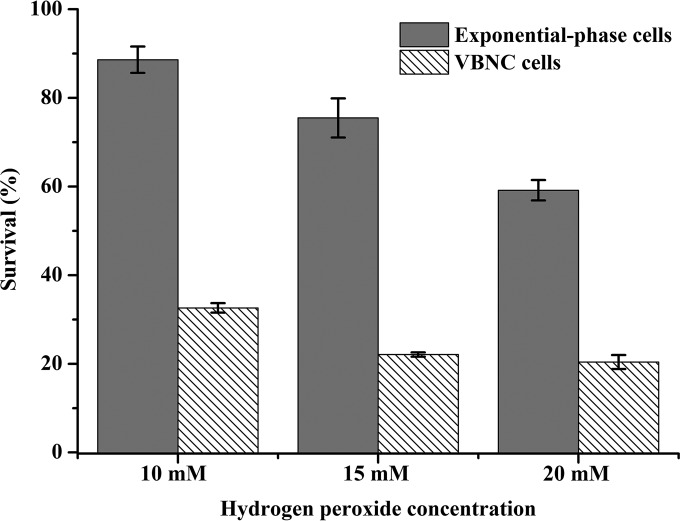
Tolerance of oxidative stress in *Escherichia coli* O157:H7 cells. Error bars represent standard deviation (SD).

The transcription level of the *z3312* gene encoding Cu, Zn-superoxide dismutase (Cu, Zn-SOD) was downregulated in the VBNC cells. Cu, Zn-SOD in the periplasm of *E. coli* catalyzes the conversion of oxygen radicals into hydrogen peroxide and oxygen (48); thus, downregulation of *z3312* would decrease the periplasmic resistance to oxidative stress upon VBNC state formation. Smith and Oliver also found that *katG* coding for periplasmic catalase was downregulated in VBNC *V. vulnificus* (49). In this study, the susceptibility of the VBNC cells to oxidative stress was investigated. As shown in Fig. 4, the survival rate of the control was higher than that of the VBNC cells during H_2_O_2_ treatment at concentrations from 10 mM to 20 mM, suggesting that the resistance of VBNC cells to oxidative stress was lower than that of the control, which was in accordance with the transcriptomic analysis. However, under conditions of H_2_O_2_ treatment from 15 mM to 20 mM, the survival rate of VBNC cells was decreased by only 7.65%, while that of the control cells was decreased by 21.62% (Fig. 4), which indicates that the oxidative tolerance of the VBNC cells was enhanced. This result was also consistent with the transcriptomic and proteomic analyses, because the ability to relieve the toxicity of reactive oxygen species (including Zwf and Gnd) was increased in the VBNC cells, which may contribute to the H_2_O_2_ tolerance. But the VBNC cells were also more susceptible to oxidative stress than the control cells. Kong et al. (6) and Masmoudi et al. (7) found that loss of culturability in *V. vulnificus* and *S. aureus* occurred concomitantly with the loss of catalase activity. Meanwhile, Serpaggi et al. (10) proposed that oxidative stress would be a driving factor for VBNC state entry. Therefore, oxidative stress caused by HPCD may induce VBNC state formation in *E. coli* O157:H7.

### Pathogenicity.

Transcript levels of *z4190* encoding SpaO and *z4195* encoding EivA were upregulated in the VBNC cells. SpaO is required for secretion of needle complex proteins of the type III secretion system (T3SS) (50), and EivA is responsible for forming a basal body of T3SS (51, 52). Upregulation of these two genes would enhance T3SS synthesis in the VBNC cells. However, downregulation of *z4194* encoding an ATP synthase for substrate recognition and energy generation for T3SS transport was detected in the VBNC cells. This would decrease the ability of T3SS to secrete virulence factors and then repress the formation of attaching and effacing (A/E) lesions on host cells. These results indicate that the pathogenicity caused by the T3SS was reduced upon VBNC state formation, although T3SS synthesis was probably enhanced. As the T3SS still exists in the VBNC cells, the pathogenicity may rebound quickly after resuscitation.

In the VBNC cells, transcript levels of *z3596* and *fimC*, which are responsible for fimbrial synthesis, were downregulated 2.50- and 2.38-fold, respectively. This result suggests that the fimbrial synthesis ability was reduced upon VBNC state entry, which would decrease the ability to adhere to host cells. Furthermore, four virulence factors were downregulated in the VBNC cells, including the *lomK* gene, the *lamB* gene, OmpA, and keto-hydroxyglutarate-aldolase. All four of these virulence factors participate in adhesion to host cells (53–56); thus, their downregulation during VBNC state entry would also decrease the adhesive ability of *E. coli* O157:H7 cells.

**FIG 5:**
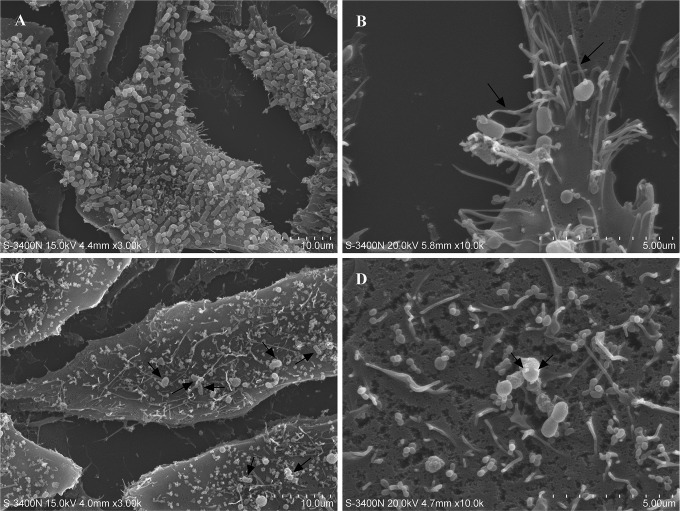
SEM image showing *Escherichia coli* O157:H7 adhering to HeLa cells. (A and B) HeLa cells adhered by the exponential-phase cells. (C and D) HeLa cells adhered by the VBNC cells. Left images, magnification of ×3,000; right images, magnification of ×10,000.

**FIG 6:**
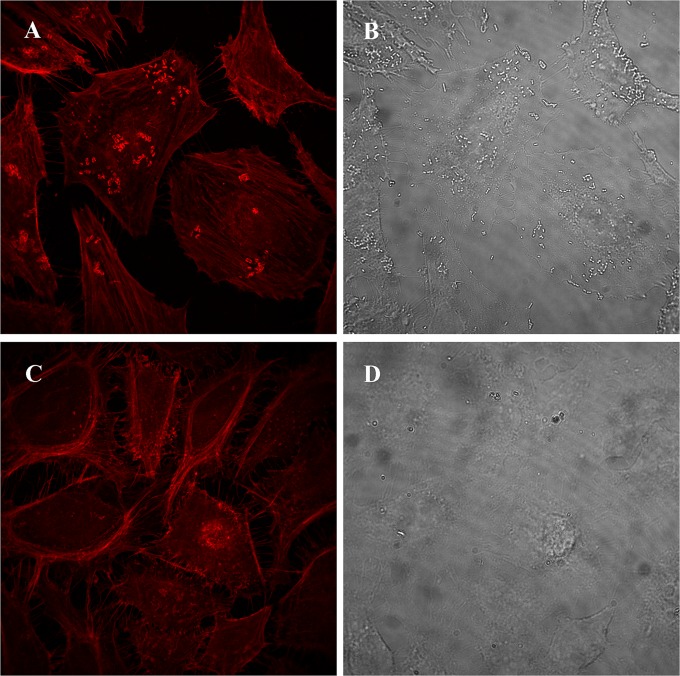
Effect of *Escherichia coli* O157:H7 on F-actin in HeLa cells. (A and B) HeLa cells infected by the exponential-phase cells. (C and D) HeLa cells infected by the VBNC cells. Left images, fluorescence micrographs; right images, phase-contrast micrographs.

**FIG 7:**
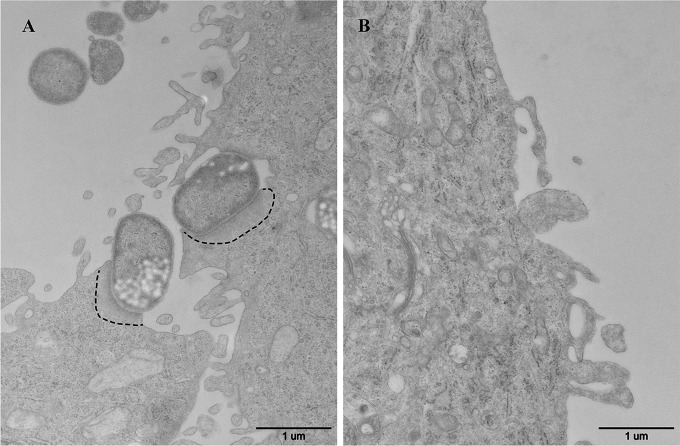
TEM image showing *Escherichia coli* O157:H7 adhering to HeLa cells. (A) HeLa cells adhered by the exponential-phase cells. Dashed lines indicate dense concentrations of microfilaments composed of F-actin. (B) HeLa cells adhered by the VBNC cells. Magnification, ×30,000.

As the pathogenicity of the VBNC cells is the most concerning issue for human health, it was further investigated by cell adhesion assay in this study. As shown in the results of scanning electron microscopy (SEM) observations, many exponential-phase cells adhered to the surface of HeLa cells (Fig. 5A), and some filamentous organelles that were 113 nm in diameter formed physical bridges between the bacteria and the infected cells (Fig. 5B, arrow). Filaments of this kind are composed of structural proteins secreted by T3SS and provide only weak attachment to host cells (57). Although the filaments spread throughout the HeLa cells (Fig. 5C) and were present on the surface of the VBNC cells (Fig. 5D, arrow), there were fewer VBNC cells adhering to the HeLa cells (Fig. 5C, arrow). These results suggest that the adhesive ability of *E. coli* O157:H7 was decreased upon VBNC state formation but that T3SS synthesis was maintained, which confirmed the transcriptomic and proteomic analyses. Then, A/E lesion formation was investigated for HeLa cells by measurement of filamentous actin (F-actin) accumulation. Intense fluorescence spots were observed on the surface of HeLa cells that had been infected with the exponential-phase cells (Fig. 6A), and the spots corresponded in size and position to adherent bacteria in comparisons with the complementary phase-contrast image (Fig. 6B). However, there were no intense fluorescence spots at the surface of HeLa cells infected by the VBNC cells (Fig. 6C), although a few adherent bacteria were seen in the complementary phase-contrast image (Fig. 6D). This phenomenon was further confirmed by TEM analysis. As shown in Fig. 7A, dense concentrations of microfilaments composed of F-actin were observed beneath attached exponential-phase cells, but no such dense structure was formed beneath attached VBNC cells (Fig. 7B). These results demonstrate that A/E lesion formation was repressed upon VBNC state formation, which confirmed the transcriptomic and proteomic analyses. Reduced pathogenicity may be an adaptation strategy for the reduced metabolic activity and the energy limitation during VBNC state formation. But it is worth noting that VBNC bacteria with reduced virulence may regain pathogenicity after resuscitation (58, 59); thus, the HPCD-induced VBNC *E. coli* O157:H7 strain would also pose a health risk to humans.

### Concluding remarks.

In this study, we explored molecular characteristics of VBNC *E. coli* O157:H7 induced by HPCD treatment by the use of RNA-seq transcriptome profiling combined with iTRAQ proteomic analysis. The differential changes in gene expression appeared to be associated with pathways contributing to VBNC state formation and VBNC cell survival at several levels. First, the membrane integrity was maintained in the VBNC cells, which functioned as a barrier protecting the cells against environmental stresses. Second, activities of central metabolic processes and gene expression were decreased and DNA replication and cell division were repressed during VBNC state formation. These changes would lead the cells to enter a state of low metabolic activity concurrently with a division arrest, i.e., the VBNC state. Third, pyruvate catabolism shifted from the TCA cycle toward the fermentative route upon VBNC state entry, leading to lower energy production. For survival, the decreased production of energy was compensated for by degradation of l-serine and l-threonine, increased generation of AMP, and enhanced electron transfer. Fourth, increased production of ammonia and NADPH and reduced CO_2_ production upon VBNC state formation could help the cells resist acid, oxidation, and high CO_2_ stresses caused by HPCD, respectively. Finally, pathogenicity was decreased in the VBNC cells, which may be an adaptation strategy against the reduced metabolic activity and the energy limitation in the cells. In conclusion, decreased metabolic activity, repressed cell division, and enhanced survival ability in *E. coli* O157:H7 might cause HPCD-induced VBNC state formation.

## MATERIALS AND METHODS

### Bacterial strain and induction of the VBNC state by HPCD treatment.

*E. coli* O157:H7 NCTC 12900 was obtained from the National Culture Type Collection (Colindale, London, United Kingdom). In accordance with the procedure described by Zhao et al. (5), VBNC *E. coli* O157:H7 was generated by HPCD treatment at 5 MPa and 25°C for 40 min. After validation by the method described by Zhao et al. (5), the HPCD-induced VBNC cells with a viable concentration of approximately 10^7^ cells/ml were harvested for both transcriptomics and proteomics experiments, and the untreated exponential-phase cells were used as the control.

### Illumina high-throughput transcriptome sequencing and data analysis.

Total RNA isolation and cDNA library construction for transcriptomic analysis were performed using the procedure described by Su et al. (13). The cDNA library was sequenced using an Illumina HiSeq 2000 genome sequencer (Illumina, San Diego, CA). After dirty raw reads were filtered, the clean reads obtained were mapped to the genome sequence of *E. coli* O157:H7 EDL933 (GenBank accession number NC_002655.2) using SOAP2, and no more than 5 mismatches were allowed. Reads mapped to rRNA or to two or more transcripts were removed from further analysis. Then, the randomness of mRNA/cDNA fragmentation (60) and the gene coverage were evaluated. In addition, the gene expression level of those uniquely mapped reads was calculated by the reads per kilobase per million (RPKM) method (61). Genes differentially expressed between the two samples were identified by the ratio of RPKMs of the two samples combined with the false-discovery rate (FDR) (62). In this study, those genes with an expression ratio greater than 2 and an FDR of <0.001 were considered to be significantly expressed (63). The transcriptomic data discussed in this study have been deposited in the NCBI’s Gene Expression Omnibus (GEO) (see below).

### iTRAQ labeling, peptide fractionation, and LC-MS/MS analysis.

Total proteins from the VBNC and control cells were extracted and quantified using methods previously described (4, 64). One hundred micrograms of protein for each sample was digested and labeled with 8-plex iTRAQ reagent (Applied Biosystems). Peptides from the VBNC cells and the control cells were labeled with reagents 113 and 114, respectively. Then, equal amounts of iTRAQ-labeled peptides were pooled, vacuum-dried, and further fractioned using an UltremexSCX column (Phenomenex) (4.6-mm inner diameter [id] by 250 mm, 5-µm-particle size). The peptides were eluted at a flow rate of 1 ml/min with a gradient of 5% buffer B (25 mM NaH_2_PO_4_, 1 M KCl, 25% ACN, pH 2.7) for 7 min, 5% to 60% buffer B for 20 min, and 60% to 100% buffer B for 2 min. The system was then maintained with 100% buffer B for 1 min before equilibration with 5% buffer B for 10 min prior to the next injection. Elution was monitored by measuring the absorbance at 214 nm, and fractions were collected every 1 min. The eluted peptides were pooled into 20 fractions. After the peptides were desalted and vacuum-dried, they were analyzed using an LC-20AD nano HPLC system (Shimadzu). Then, the eluted peptides were subjected to MS analysis using a TripleTOF 5600 system (AB Sciex). The MS was operated with a resolving power (RP) of ≥30,000 (full width at half-maximum [FWHM]) for time of flight (TOF) MS scans. For IDA, survey scans were acquired in 250 ms, and as many as 30 product ion scans were collected if data exceeded a threshold of 120 cps and showed a 2+ to 5+ charge state. The total cycle time was fixed to 3.3 s. The Q2 transmission window was 100 Da for 100%. Four time bins were summed for each scan at a pulser frequency value of 11 kHz through monitoring of the 40-GHz multichannel TDC detector with four-anode channel detection. A sweeping collision energy setting of 35 ± 5 eV coupled with iTRAQ-adjusted rolling collision energy was applied to all precursor ions for collision-induced dissociation. Dynamic exclusion was set for 1/2 of the peak width (15 s), and then the precursor was refreshed off the exclusion list.

### iTRAQ protein identification and quantification.

Raw data files acquired from the Orbitrap were converted into MGF files, which were searched against the protein translation database of *E. coli* O157:H7 EDL933 containing 5,397 sequences by the use of the MASCOT search engine (Matrix Science, London, United Kingdom; version 2.3.02). For protein identification, a mass tolerance of 0.05 Da was permitted for intact peptide masses and a mass tolerance of 0.1 Da for fragmented ions, with allowance for one missed cleavage in the trypsin digests, Gln→pyro-Glu (N-terminal Q), methionine oxidation, and iTRAQ (Y) as potential variable modiﬁcations and carbamidomethyl modification of cysteine, iTRAQ (N terminus), and iTRAQ (K) as fixed modiﬁcations. The charge states of peptides were set to +2 and +3. Specifically, an automatic decoy database search was performed in Mascot by choosing the decoy checkbox in which a random sequence of database is generated and tested for raw spectra as well as the real database. To reduce the probability of false peptide identification, only peptides with results at the 95% confidence interval that were greater than “identity” as determined by a Mascot probability analysis were counted as identified. Also, each protein identification with an acceptable confidence result involved at least one unique peptide. For protein quantitation, it was required that a protein contained at least two unique peptides. The quantitative protein ratios were weighted and normalized by use of the median ratio in Mascot. Those proteins with ratio fold changes of >1.2 and *P* values of <0.05 were considered to be significantly differentially expressed (65, 66). Functional annotations of the proteins were conducted using the Blast2GO program against the nonredundant protein database (NR; NCBI). The KEGG database (http://www.genome.jp/kegg/) and the COG database (http://www.ncbi.nlm.nih.gov/COG/) were used to classify and group the identified proteins.

### Investigation of VBNC state formation under conditions of HPCD treatment by homologous expression.

*z2046* and *ompF* genes were amplified by PCR from *E. coli* O157:H7 chromosomal DNA using the corresponding primers (see Table S5 in the supplemental material). After digestion by NcoI and KpnI, the PCR amplicon was inserted into expression vector pTrcHisA (Invitrogen). The recombinant plasmids were transformed into *E. coli* O157:H7 by electroporation and then analyzed by sequencing. The two recombinant strains harboring pTrcHiA-*z2046* and pTrcHiA-*ompF* were designated *E. coli* pTrc-*z2046* and *E. coli* pTrc-*ompF*, respectively. Meanwhile, the control strain (*E. coli* pTrcHiA) was constructed by introducing empty vector pTrcHisA into *E. coli* O157:H7.

Two hundred microliters of an overnight culture of the control strain or the recombinant strain was inoculated into 20 ml of tryptic soy broth (TSB) containing 100 µg/ml ampicillin and incubated at 37°C with shaking at 200 rpm to an optical density at 600 nm (OD_600_) of 0.4. Then, IPTG was added to the culture to reach a final concentration of 1 mM and the reaction mixture was further incubated at 16°C with shaking at 200 rpm to an OD_600_ of 1.0. Tricine–SDS-PAGE analysis was used to investigate the expression of *z2046*, and the expression of *ompF* was examined by SDS-PAGE analysis. Meanwhile, overproduced bands were cut from the gels and further identified by LC-MS/MS analysis. To investigate the functional roles of these overproduced proteins in VBNC state formation induced by HPCD treatment, the control and recombinant strains subjected to IPTG induction were treated by the use of HPCD at 5 MPa and 25°C for 15 min, and the percentage of VBNC cells for each HPCD-treated strain was calculated (5).

### Hydrogen peroxide challenge.

Resistance to oxidative stress was assessed using 0.01 M phosphate-buffered saline (PBS; pH 7.4) with 10 mM, 15 mM, or 20 mM H_2_O_2_, which was sterilized using a filter before the tolerance assay was performed. The VBNC cells and the control cells were washed with PBS and then resuspended in 5 ml of different PBS-H_2_O_2_ solutions to achieve a cell concentration of 10^8^ CFU/ml. The cell suspensions were incubated statically at 37°C for 1 h. After that, samples were immediately centrifuged at 8,000 × *g* for 10 min at room temperature, and the pellets were washed once with PBS and then resuspended in 5 ml of 0.85% NaCl solution. The percentage of viable cells was measured using a flow cytometer (BD Immunocytometry Systems, San Jose, CA, USA) after the cells were stained using a Live/Dead *Bac*Light bacterial viability kit (Molecular Probes Inc., Eugene, OR) (5).

### Fluorescence anisotropy measurements.

Membrane fluidity was determined by measuring fluorescence anisotropy according to the method described by Chu-Ky et al. (67). After incubation of the cells with 2 mM 1,6-diphenyl-1,3,5-hexatriene (Sigma-Aldrich), fluorescence anisotropy of the VBNC and control cells was determined by the use of a spectrofluorimeter (F-7000; Hitachi, Japan) with a 352-nm excitation wavelength and a 402-nm emission wavelength.

### Adhesion assay.

HeLa cells (Cell Resource Center, CAMS/PUMC, China) were cultured in high-glucose Dulbecco’s modified Eagle’s medium (DMEM; HyClone) supplemented with 10% fetal bovine serum (FBS; Gibco) and 1% penicillin-streptomycin (Sigma-Aldrich) in a humidified 5% CO_2_ balanced air incubator (Thermo Fisher Scientific) at 37°C. One milliliter of exponential-phase HeLa cells suspended in DMEM–10% FBS without antibiotics was seeded into each well of a 24-well plate containing a 13-mm-diameter glass coverslip. HeLa cells were grown to 70% to 90% confluence and washed with PBS, and then 1 ml of the VBNC cells or the control cells resuspended in DMEM–10% FBS with a concentration of 5 × 10^8^ cells/ml was added to each well and the reaction mixture was incubated for 6 h. To minimize extracellular multiplication of the infecting bacteria, infected HeLa cells were washed with PBS after incubation for 3 h and incubated for another 3 h in fresh DMEM–10% FBS. For SEM and TEM, infected HeLa cells were fixed with 3% glutaraldehyde for 24 h. The SEM observation was carried out with a Hitachi S-3400 N SEM (Hitachi Instruments Inc., Japan), and the TEM observation was performed with a JEM-1230 TEM (JEOL, Japan Electronics Co., Ltd., Japan). In order to observe F-actin accumulation, infected HeLa cells were stained with tetramethylrhodamine B isothiocyanate-phalloidin (Sigma-Aldrich) according to the manufacturer’s instructions. The samples were examined by confocal laser scanning microscopy (Nikon Corporation, Tokyo, Japan), and fluorescence and phase-contrast micrographs of the same field were recorded.

### Statistical analysis.

Analysis of variance (ANOVA) was performed by using OriginPro 8.5 software (OriginLab Corporation, Northampton, MA). The significance level was 0.05.

### Accession number.

The transcriptomic data discussed in this study have been deposited in the NCBI Gene Expression Omnibus (GEO) and are accessible through GEO Series accession number GSE62394 (http://www.ncbi.nlm.nih.gov/geo/query/acc.cgi?acc=GSE62394).

## SUPPLEMENTAL MATERIAL

Figure S1  Distribution statistics of *Escherichia coli* O157:H7 reads mapped to the reference genes. (A) The VBNC cells. (B) The exponential-phase cells. Download Figure S1, PDF file, 0.1 MB

Figure S2  Gene coverage statistics of transcripts for *Escherichia coli* O157:H7. (A) The VBNC cells. (B) The exponential-phase cells. Download Figure S2, PDF file, 0.4 MB

Figure S3  Molecular weight distribution of total identified proteins for *Escherichia coli* O157:H7 using the iTRAQ platform. Download Figure S3, PDF file, 0.5 MB

Figure S4  COG analysis of total identified proteins for *Escherichia coli* O157:H7 using the iTRAQ platform. Download Figure S4, PDF file, 0.4 MB

Figure S5  Membrane fluidity of *Escherichia coli* O157:H7 cells. Error bars represent standard deviation. Download Figure S5, PDF file, 0.1 MB

Table S1  Summary of RNA-Seq data for *Escherichia coli* O157:H7 in the VBNC state and the exponential phase mapped to the reference genome. Table S1, PDF file, 0.03 MB

Table S2  Differentially expressed genes in the VBNC *Escherichia coli* O157:H7 cells induced by high-pressure CO_2_ Table S2, PDF file, 0.04 MB

Table S3  Summary of protein identification for the VBNC and exponential-phase *Escherichia coli* O157:H7 cells using the iTRAQ platform. Table S3, PDF file, 0.01 MB

Table S4  Differentially expressed proteins in the VBNC *Escherichia coli* O157:H7 cells induced by high-pressure CO_2_ Table S4, PDF file, 0.04 MB

Table S5  Oligonucleotide primers used in this study. Table S5, PDF file, 0.01 MB
