# Supplementary figures and images for: New Insights into the Formation of Viable but Nonculturable Escherichia coli O157:H7 Induced by High-Pressure CO2
Source: mBio. 2016 Aug 30;7(4):e00961-16. doi: 10.1128/mBio.00961-16 (PMC4999544; doi:10.1128/mBio.00961-16)

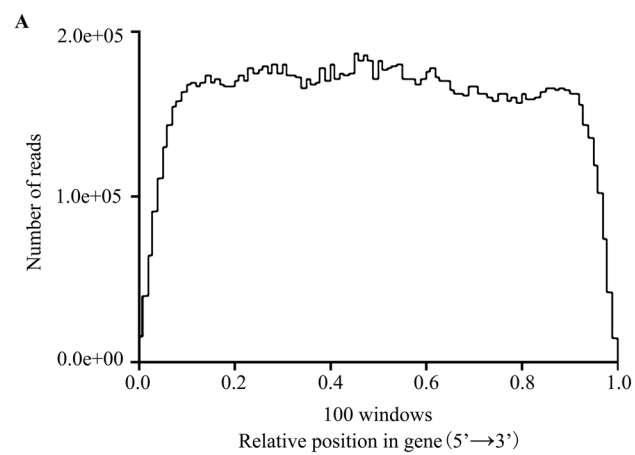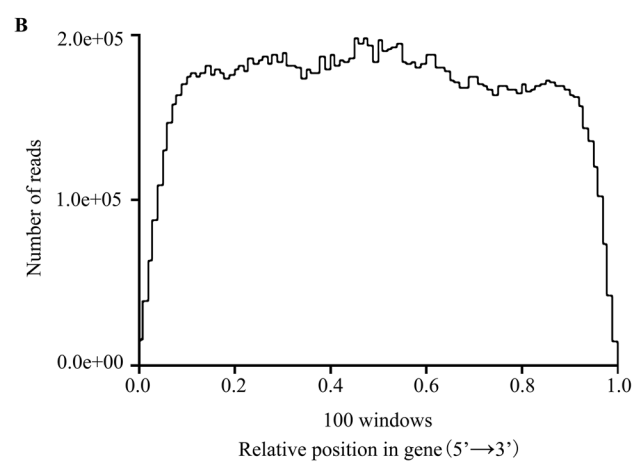

Supplement: Figure S1 — Distribution statistics of Escherichia coli O157:H7 reads mapped to the reference genes. (A) The VBNC cells. (B) The exponential-phase cells. Download [file mbo004162960sf1.pdf]

**A**

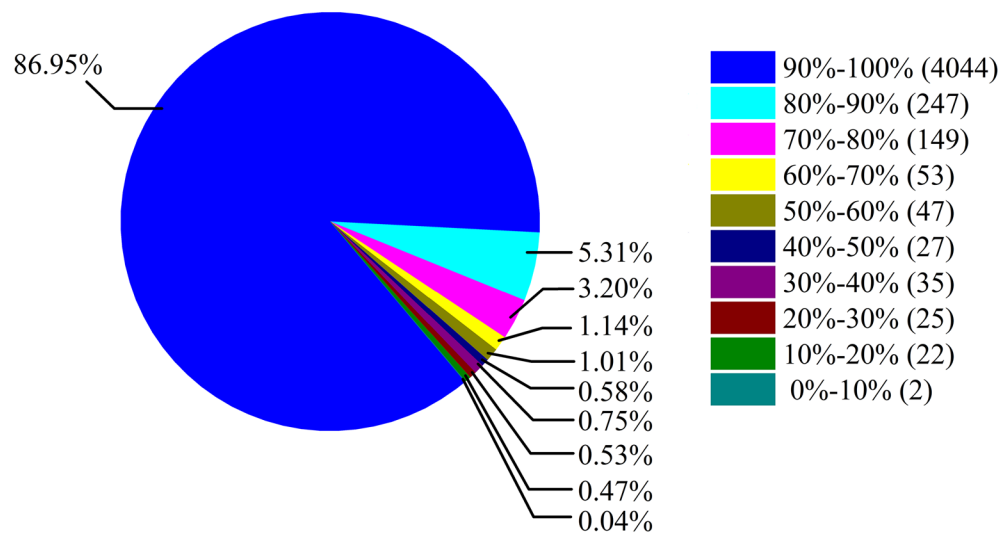

**B**

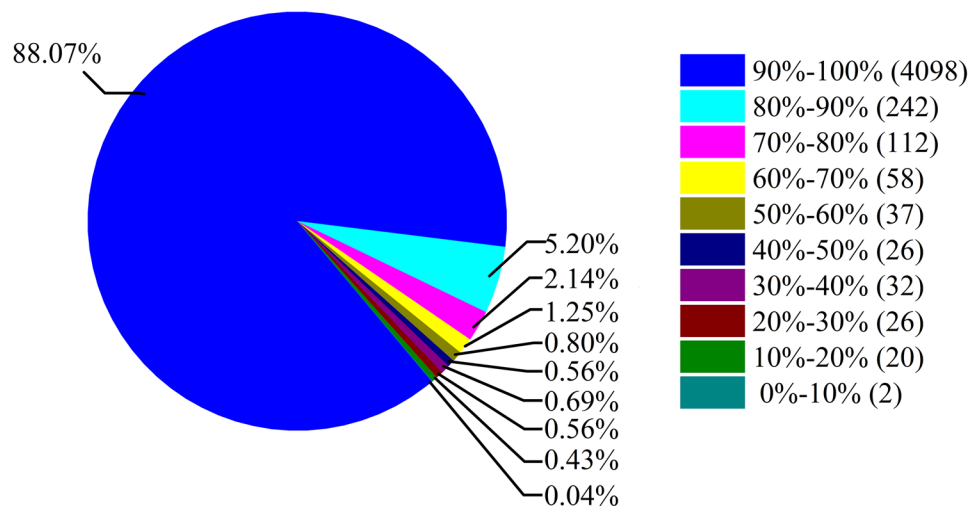

Supplement: Figure S2 — Gene coverage statistics of transcripts for Escherichia coli O157:H7. (A) The VBNC cells. (B) The exponential-phase cells. Download [file mbo004162960sf2.pdf]

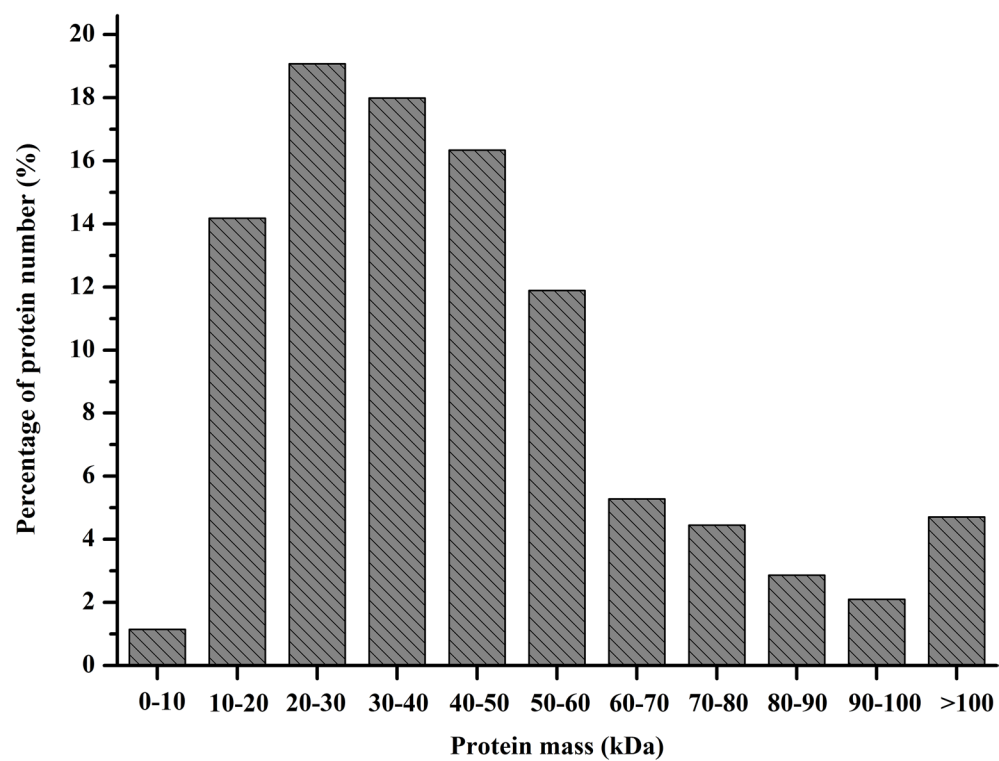

Supplement: Figure S3 — Molecular weight distribution of total identified proteins for Escherichia coli O157:H7 using the iTRAQ platform. Download [file mbo004162960sf3.pdf]

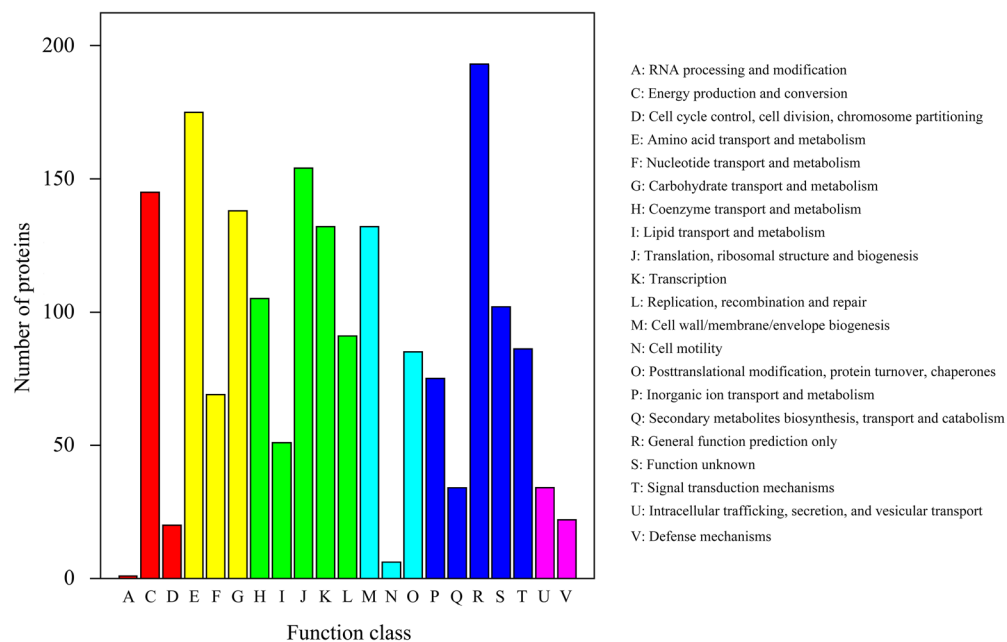

Supplement: Figure S4 — COG analysis of total identified proteins for Escherichia coli O157:H7 using the iTRAQ platform. Download [file mbo004162960sf4.pdf]

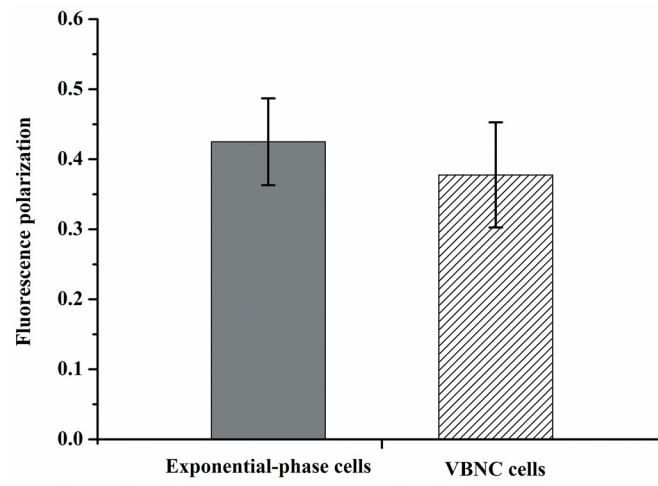

Supplement: Figure S5 — Membrane fluidity of Escherichia coli O157:H7 cells. Error bars represent standard deviation. Download [file mbo004162960sf5.pdf]
