# Supplementary material for: New Insights into the Formation of Viable but Nonculturable Escherichia coli O157:H7 Induced by High-Pressure CO2
Source: mBio. 2016 Aug 30;7(4):e00961-16. doi: 10.1128/mBio.00961-16 (PMC4999544; doi:10.1128/mBio.00961-16)
Supplement: Table S1 — Summary of RNA-Seq data for Escherichia coli O157:H7 in the VBNC state and the exponential phase mapped to the reference genome. [file mbo004162960st1.pdf]

Table S1. Summary of RNA-Seq data for *Escherichia coli* O157:H7 in the VBNC state and the exponential phase mapped to the reference genome.

Category	Number of reads for the VBNC cells	Number of reads for the exponential-phase cells
Total reads	25674506	25707052
Total basepairs	2310705540	2313634680
Total mapped reads	24975882	24944837
Perfect match	20399683	20749212
≤ 5 bp mismatch	4576199	4195625
Unique match	24434903	23634364
Multi-position match	540979	1310473
Total unmapped reads	698624	762215
