# Supplementary material for: New Insights into the Formation of Viable but Nonculturable Escherichia coli O157:H7 Induced by High-Pressure CO2
Source: mBio. 2016 Aug 30;7(4):e00961-16. doi: 10.1128/mBio.00961-16 (PMC4999544; doi:10.1128/mBio.00961-16)
Supplement: Table S2 — Differentially expressed genes in the VBNC Escherichia coli O157:H7 cells induced by high-pressure CO2 [file mbo004162960st2.pdf]

Table S2. Differentially expressed genes in the VBNC *Escherichia coli* O157:H7 cells induced by high pressure CO_2.

Gene and functional category	Gene ID[a]	Function[b]	Fold change[c]
**Carbohydrate transport and metabolism**			
srlA_1	958148	PTS system, glucitol/sorbitol-specific IIC component	0.38
z4200	958343	Glycosyl hydrolase, family 15 domain protein	0.29
z4201	958344	Glycosyl hydrolase, family 15 domain protein	0.27
agaD	958600	PTS system, galactosamine-specific IID component	0.40
ulaA	959930	PTS system, ascorbate-specific IIC component	0.45
malM	960086	Maltose operon periplasmic protein	0.46
lamB	960087	Maltoporin	0.42
**Amino acid transport and metabolism**			
fixA	956710	Electron transfer flavoprotein β subunit	0.33
fixX	956715	Ferredoxin like protein	0.21
metN	956944	D-methionine transport system ATP-binding protein	0.43
artJ	958254	Arginine transport system substrate-binding protein	0.41
z4464	958585	L-serine deaminase	2.30
argI	959783	Ornithine carbamoyltransferase	0.23
metA	960117	Homoserine O-succinyltransferase	0.43
argC	960174	N-acetyl-gamma-glutamyl-phosphate reductase	0.39
metF	960190	Methylenetetrahydrofolate reductase	0.39
metR	960330	Regulator for metE and metH	0.40
yhaR	961449	TdcF protein	2.16
**Nucleotide metabolism**			
z0404	957221	Transcriptional activator of the allD operon	0.42
z0661	957497	Tartronate-semialdehyde synthase	0.31
z0662	957498	Hydroxypyruvate isomerase	0.31
z0663	957501	2-hydroxy-3-oxopropionate reductase	0.29
z0665	957507	Allantoin permease	0.35
z0666	957508	Allantoinase	0.38
z0667	957509	Allantoinase	0.39
**DNA recombination**			
z0953	957934	Protein NinG	2.21
z1866	959822	Integrase of prophage CP-933X	0.29
z5490	960196	Insertion element IS1 protein InsB	0.45
intC	960221	Putative prophage integrase	0.36
z2101	960570	Crossover junction endonuclease RusA	0.16
z2981	961893	Putative IS629 transposase	0.39

intU	962033	Integrase for prophage CP-933U	0.47
**Transcription and translation**			
z3345	956970	Antitermination protein Q	0.13
z3357	957015	Regulatory protein CII	0.29
ybcH	957552	N5-glutamine methyltransferase	0.40
hyaD	959038	Hydrogenase 1 maturation protease	0.49
**Cell division**			
z1876	959832	Lysozyme	0.37
z2046	960518	DNA-binding transcriptional regulator DicC	0.050
z2371	961111	Lysozyme	0.15
**Stress response**			
z3312	957692	Cu/Zn superoxide dismutase	0.49
ecnB	959974	Entericidin B	3.14
hdeA	961158	Acid-resistance protein HdeA	2.11
hdeB	961159	Acid-resistance protein HdeB	2.52
yedU	961970	Molecular chaperone Hsp31	0.43
**Electron transfer chain**			
ccmD	957172	Heme exporter protein D	2.21
z2702	961645	Ferredoxin-like protein YdhY	2.10
yodB	961979	Cytochrome b561	2.41
**Membrane transport**			
ylcB	957569	Cu(I)/Ag(I) efflux system outer membrane protein CusC	2.98
ybdA	957603	MFS transporter, enterobactin (siderophore) exporter	0.44
z5415	960280	Sulfite exporter TauE/SafE family protein	0.43
z2185	960646	Protein YneE	0.32
z4357	961277	Biopolymer transport protein ExbD	3.66
z2503	961357	MFS transporter, bicyclomycin/chloramphenicol resistance protein	0.34
**Pathogenicity**			
z3596	957046	Minor fimbrial subunit	0.40
z4194	957284	ATP synthase in type III secretion protein N	0.29
z3276	957303	Fimbrial protein	0.44
lomK	957976	Putative virulence related protein PagC	0.26
z4190	958331	Type III secretion protein SpaO	2.78
z4195	958340	Type III secretion protein EivA	2.13
fimC	959737	Fimbrial chaperone protein	0.42
**Minerals transport and metabolism**			
ssuC	958915	Sulfonate transport system permease protein	0.20
ycbN	958916	Alkanesulfonate monooxygenase	0.43

fhuF	959686	Ferric iron reductase protein FhuF	0.31
phnC	960018	Phosphonate transport system ATP-binding protein	0.41
phnK	960026	Putative phosphonate transport system ATP-binding protein	0.43
phnL	960027	Putative phosphonate transport system ATP-binding protein	0.47
phnP	960031	Carbon-phosphorus lyase complex accessory protein	0.35
**RNA degradation**			
z0284	957038	mRNA interferase YafQ	0.49
z1678	959374	Putative uncharacterized protein YmdA	0.36
**Protein degradation**			
z3651	957594	Aminopeptidase	2.86
**Cell motility**			
z3672	958232	FlxA-like family protein	0.38
z5895	959754	Chemotaxis protein MotB	2.08
fliG	961939	Flagellar motor switch protein FliG	0.45
fliH	961940	Flagellar assembly protein FliH	3.06
**Fuction prediction only**			
z0974	957963	Tail component of prophage CP-933K	0.35
z3313	958214	Tail component of prophage CP-933V	0.44
z4084	958231	Alkyldihydroxyacetonephosphate synthase	0.35
ybjW	958391	Hydroxylamine reductase	0.34
z1554	959190	Putative ABC transport system permease protein	0.38
yibD	960931	Glycosyltransferase	2.15
**Fuction unknown**			
z3341	956966	Hypothetical protein	2.09
z3400	957105	Hypothetical protein	2.15
z0965	957950	Hypothetical protein	0.47
z1340	958985	Hypothetical protein	0.41
yjfL	959939	Putative membrane protein	3.92
yjcB	960064	Hypothetical protein	0.46
ygbF	960428	Hypothetical protein	0.45
z2100	960569	Hypothetical protein	0.36
z2240	960705	Leucine Rich Repeat family protein	9.93
z2241	960706	Leucine Rich Repeat family protein	5.05
z5161	960801	Hypothetical protein	0.21
yibG	960956	Hypothetical protein	0.20
z2309	960996	Hypothetical protein	0.34
z2311	960998	Hypothetical protein	0[d]
z2695	961638	Hypothetical protein	0.43

z2717	961660	Hypothetical protein	0.47
z2774	961712	Hypothetical protein	0.47

[a]Gene ID was from the NCBI database.

[b]Functions were assigned from the KEGG pathways for *Escherichia coli* O157:H7 EDL933.

[c]Changed expression levels in VBNC cells compared to those in the exponential-phase cells.

[d]There was no gene expression in VBNC cells.
