# Supplementary material for: New Insights into the Formation of Viable but Nonculturable Escherichia coli O157:H7 Induced by High-Pressure CO2
Source: mBio. 2016 Aug 30;7(4):e00961-16. doi: 10.1128/mBio.00961-16 (PMC4999544; doi:10.1128/mBio.00961-16)
Supplement: Table S3 — Summary of protein identification for the VBNC and exponential-phase Escherichia coli O157:H7 cells using the iTRAQ platform. [file mbo004162960st3.pdf]

Table S3. Summary of protein identification for the VBNC and exponential-phase *Escherichia coli* O157:H7 using the iTRAQ platform.

Category	Identified number
Total spectra	241021
Spectra	22206
Unique spectra	22165
Peptide	7215
Unique peptide	7194
Protein	1573
