# Supplementary material for: New Insights into the Formation of Viable but Nonculturable Escherichia coli O157:H7 Induced by High-Pressure CO2
Source: mBio. 2016 Aug 30;7(4):e00961-16. doi: 10.1128/mBio.00961-16 (PMC4999544; doi:10.1128/mBio.00961-16)
Supplement: Table S4 — Differentially expressed proteins in the VBNC Escherichia coli O157:H7 cells induced by high-pressure CO2 [file mbo004162960st4.pdf]

Table S4. Differentially expressed proteins in the VBNC *Escherichia coli* O157:H7 cells induced by high pressure CO_2.

Protein and functional category	Accession number[a]	Gene	Fold change[b]
**Carbohydrate transport and metabolism**			
Pyruvate dehydrogenase subunit E1	NP_285810.1	*aceE*	1.51
Aconitate hydratase 2	NP_285814.1	*acnB*	0.72
Type II citrate synthase	NP_286436.1	*gltA*	1.85
D-lactate dehydrogenase	NP_287766.1	*ldhA*	1.53
Glucose-6-phosphate dehydrogenase	NP_288289.1	*zwf*	1.62
6-phosphogluconate dehydrogenase	NP_288534.1	*gnd*	1.33
β-D-glucoside glucohydrolase	NP_288709.1	*bglX*	0.56
D-lactate dehydrogenase	NP_288710.1	*dld*	0.21
Phosphoenolpyruvate-protein phosphotransferase	NP_288978.1	*ptsI*	0.61
Maltose ABC transporter ATP-binding protein	NP_290669.1	*malK*	2.40
**Amino acid transport and metabolism**			
Threonine synthase	NP_285696.1	*thrC*	2.23
Lysine-, arginine-, ornithine-binding periplasmic protein	NP_288884.1	*argT*	2.22
D-3-phosphoglycerate dehydrogenase	NP_289481.1	*serA*	1.49
**Nucleotide metabolism**			
Carbamoyl phosphate synthase large subunit	NP_285727.1	*carB*	0.59
Adenylate kinase	NP_286215.1	*adk*	1.34
Ribonucleoside-diphosphate reductase subunit α	NP_288808.1	*nrdA*	2.14
Inosine 5'-monophosphate dehydrogenase	NP_289062.1	*guaB*	0.41
Phosphoribosylformylglycinamidine synthase	NP_289113.1	*purL*	0.60
Ribokinase	NP_290391.1	*rbsK*	0.64
Adenylosuccinate synthetase	NP_290807.1	*purA*	1.60
**DNA replication**			
DNA polymerase III subunit α	NP_285878.1	*dnaE*	0.36
Replication initiation regulator SeqA	NP_286402.1	*seqA*	1.76
**Transcription and translation**			
Isoleucyl-tRNA synthetase	NP_285720.1	*ileS*	1.48
Glutaminyl-tRNA synthetase	NP_286394.1	*glnS*	2.12
30S ribosomal protein S1	NP_286786.1	*rpsA*	0.79
DNA topoisomerase I	NP_287925.1	*topA*	1.88
Transcription elongation factor GreA	NP_289755.2	*greA*	1.64
30S ribosomal protein S4	NP_289857.1	*rpsD*	1.39
30S ribosomal protein S5	NP_289864.1	*rpsE*	0.75
50S ribosomal protein L24	NP_289870.1	*rplX*	1.55
Glycyl-tRNA synthetase subunit α	NP_290144.1	*glyQ*	0.33
Fic family protein	NP_290168.1	*z5009*	0.25

**Cell division**			
DamX protein	NP_289927.1	*damX*	1.85
**Stress response**			
Heat shock protein 90	NP_286214.1	*htpG*	1.51
Glucan biosynthesis protein D	NP_287736.1	*mdoD*	0.56
Molecular chaperone GroEL	NP_290776.1	*groEL*	1.24
**Electron transfer chain**			
Glutamate-1-semialdehyde aminotransferase	NP_285850.1	*hemL*	1.99
**Membrane biosynthesis and transport**			
Outer membrane protein F	NP_286804.1	*ompF*	1.71
UTP-glucose-1-phosphate uridylyltransferase	NP_287481.1	*galU*	2.10
sn-glycerol-3-phosphate dehydrogenase subunit A	NP_288817.1	*glpA*	0.43
Undecaprenyl phosphate 4-deoxy-4-formamido-L-arabinose transferase	NP_288830.1	*z3512*	0.27
Aminoglycoside/multidrug efflux system	NP_289022.1	*acrD*	0.22
D-arabinose 5-phosphate isomerase	NP_289257.2	*gutQ*	0.33
NAD(P)H-dependent *sn*-glycerol-3-phosphate dehydrogenase	NP_290191.1	*gpsA*	1.59
**Pathogenicity**			
Outer membrane protein A	NP_286832.1	*ompA*	0.79
Keto-hydroxyglutarate-aldolase	NP_288287.1	*eda*	0.67
**RNA degradation**			
Exoribonuclease R	NP_290809.2	*vacB*	0.44
**Protein degradation**			
Isoaspartyl dipeptidase	NP_290945.1	*iadA*	0.60
**Coenzyme metabolism**			
Thiamine biosynthesis protein ThiI	NP_286165.1	*yajK*	0.38
Lipoyl synthase	NP_286354.1	*lipA*	0.68
**Fuction prediction only**			
Phage inhibition, colicin resistance and tellurite resistance protein	NP_286707.1	*terA*	0.31
Outer membrane protein assembly complex subunit YfiO	NP_289150.1	*z3889*	0.47
ABC transporter ATP-binding protein	NP_289900.1	*yheS*	0.28
**Function unknown**			
Hypothetical protein	NP_285859.1	*yaeH*	2.26
Hypothetical protein	NP_288049.1	*ydgA*	0.48
Hypothetical protein	NP_289066.1	*z3776*	2.34

[a]Accession number was from the NCBI database.

[b]Changed expression levels in VBNC cells compared to those in the exponential-phase cells.
