# Supplementary material for: New Insights into the Formation of Viable but Nonculturable Escherichia coli O157:H7 Induced by High-Pressure CO2
Source: mBio. 2016 Aug 30;7(4):e00961-16. doi: 10.1128/mBio.00961-16 (PMC4999544; doi:10.1128/mBio.00961-16)
Supplement: Table S5 — Oligonucleotide primers used in this study. [file mbo004162960st5.pdf]

Table S5. Oligonucleotide primers used in this study.

Primer	Sequence (5’-3’)
Z2046-F	CATGCCATGGGATTGAAAATTGATGCTATAG
Z2046-R	GGGGTACCGTTCAGTGGTGATTTTCATTGT
OmpF-F	CATGCCATGGGAATGAAGCGCAATATTCTG
OmpF-R	GGGGTACCGCTATTAGAACTGGTAAACGAT

Underlined sequences CCATGG and GGTACC represent restriction sites *Nco*I and *Kpn*I, respectively.
